# Participation in Clinic-Based Referral and Navigation Services Among Families With Social Needs

**DOI:** 10.1001/jamanetworkopen.2025.0056

**Published:** 2025-02-28

**Authors:** Abigail Seide, Omolara Thomas Uwemedimo, Rehana Rasul, Caren Steinway, Michelle Katzow, Sophia Jan, Eun Ji Kim

**Affiliations:** 1 Northwell Health, New Hyde Park, New York; 2 Department of Pediatrics, Cohen Children’s Medical Center, Northwell Health, New Hyde Park, New York; 3 Strong Children Wellness Medical Group, Jamaica, New York; 4 Department of Epidemiology and Biostatistics, Graduate School of Public Health and Health Policy, City University of New York, New York, New York; 5 Feinstein Institutes for Medical Research, Northwell Health, Manhasset, New York; 6 Department of Medicine, Zucker School of Medicine at Hofstra/Northwell, Manhasset, New York

## Abstract

**Importance:**

Social determinants of health (SDOH) are associated with health outcomes. Thus, providing referrals for unmet social needs within clinical settings may improve the health of children.

**Objective:**

To examine the prevalence and demographic characteristics of pediatric families with unmet social needs and their association with families accepting help from a pediatric clinical practice.

**Design, Setting, and Participants:**

This cross-sectional study examined data from a comprehensive SDOH screening and referral program at a large academic pediatric practice in the US. Participants were caregivers of patients younger than 17 years and indicated at least 1 social need. Data were collected from April 16, 2018, through September 29, 2019, and analyzed from November 18, 2019, through December 17, 2019. The data review was finalized December 20, 2023.

**Exposure:**

Screening for SDOH in primary care.

**Main Outcome and Measures:**

The main outcome was interest in assistance among patient families with at least 1 social need. Factor analyses categorized social needs as basic needs, stress, challenges to economic mobility, and marginalization. χ^2^ Tests and multivariable Poisson regression were used to examine the associations between patient characteristics and caregiver interest in assistance.

**Results:**

There were 758 caregivers (median [IQR] age, 34 [29-40] years; 614 mothers [81.0%]) reporting at least 1 unmet social need, such as food or utility insecurity or unemployment. The median age of children was 23 months (IQR, 4-70 months), and 163 (21.5%)were of Asian, Pacific Islander, or Native Hawaiian ; 213 (28.1%) of Black; 156 (20.6%) of Latino or Hispanic; 37 (4.9%) of White ; and 122 (16.1%) of other race and ethnicity. A total of 315 caregivers (41.6%) were not interested in assistance. Families with basic needs (adjusted prevalence ratio [PR], 5.56; 95% CI, 3.33-10.00), stress (adjusted PR, 1.75; 95% CI, 1.43-2.17), challenges to economic mobility (adjusted PR, 2.17; 95% CI, 1.67-2.86), or marginalization (adjusted PR, 1.41; 95% CI, 1.15-1.72) were more likely to be interested in assistance. Additionally, Black race (adjusted PR, 1.23; 95% CI, 1.01-1.49), other race and ethnicity (adjusted PR, 1.22; 95% CI, 1.01-1.47), and inadequate social support (adjusted PR, 1.85; 95% CI, 1.32-2.63) were associated with acceptance of referral services.

**Conclusions and Relevance:**

These findings suggest that the implementation of referral programs may help to achieve health equity, especially among marginalized populations, and improve the referral process for families who have social needs but are not interested in assistance.

## Key Points

**Question:**

What patient characteristics are associated with caregivers’ interest in assistance in meeting social needs from a general pediatric practice?

**Findings:**

In this cross-sectional study of 758 patient families with at least 1 unmet social need, 315 (41.6%) were not interested in receiving assistance. Child race and ethnicity and caregiver education, as well as having tangible social needs, were associated with accepting assistance.

**Meaning:**

These findings suggest that there are opportunities to improve communication to patient families about receiving assistance for a variety of social needs at their child’s pediatric clinic.

## Teaser

This cross-sectional study examines the prevalence of unmet social needs and characteristics associated with accepting clinic-based referral and navigation services among family receiving care from a large pediatric practice.

## Introduction

Screening for social needs in clinic-based settings has been an emerging focus for quite some time.^1,2,3^ As more hospitals and health systems implement social determinants of health (SDOH) screening and referral programs,^4^ it is important to assess participation in programs to help patient families with social needs to access referrals and navigation to address those needs. Moreover, it is necessary to evaluate which, if any, patient characteristics are associated with the family’s interest in the assistance of social resources, which is especially important as racial and ethnic minority groups are less likely to access preventive care^5,6^ and may face additional barriers to receiving this care.

Social determinants of health are largely responsible for health inequities, accounting for as much as 80% of health outcomes.^7^ Examples of health inequities are differences in length of life; quality of life; rates of disease, disability, and mortality; severity of disease; and access to treatment,^8,9^ which are influenced by SDOH generally categorized as genetics, behavior, environmental and physical influences, medical care, and social factors.^10^ Both patients and physicians agree that patients’ social needs are as important to address as their medical conditions.^11,12,13^ National organizations, such as the American Academy of Pediatrics, have made recommendations to address social needs as part of routine child care.^14^ The Centers for Medicare & Medicaid Services has recently placed an emphasis on screening and addressing social needs across all of its programs.^15^

The provision of navigation for referrals for unmet social needs has been shown to increase the likelihood of families using resources, including diverse immigrant families and families with limited English proficiency.^16^ When patients screen positive for unmet social needs, navigation is provided by connecting them with resources or referrals. There is also evidence that navigation for unmet social needs can be beneficial for child and family well-being.^3^ In addition, primary care–based screening and referral systems for unmet basic needs have increased families’ receipt of community-based resources, such as local food banks, shelters for unhoused individuals, and a medical-legal partnership program.^17^ Despite the benefit of using social services to address social needs, families frequently are not interested in the navigation services offered.^17^ Social support or social capital has also been shown to be associated with patients’ health.^18,19,20^ Some examples are educational attainment being influenced by health in adolescence,^18^ investment in social capital being associated with population-level mortality,^21^ and social capital relating to improved health care access.^20^

Despite unmet needs, many patients who screen positive for social needs subsequently decline referral services to address those needs. Therefore, examination of demographic factors associated with interest in accepting navigation services for unmet social needs is important in achieving equitable health. The aim of our study was to examine the prevalence and associated factors of acceptance of assistance among caregivers with unmet social needs within a pediatric practice–based SDOH screening and referral program. We compared patient families’ demographics and their likelihood to be interested in assistance, whether the likelihood to be interested in assistance is associated with a type of unmet social need, and whether social support has any interaction with interest in assistance.

## Methods

### Study Design, Setting, and Population

This cross-sectional analysis of an SDOH screening and referral program collected data from April 16, 2018, through September 29, 2019. The SDOH screening was conducted at a large, academic hospital–based general pediatric ambulatory practice that serves patients from all New York City and Long Island counties. We included families who reported having at least 1 unmet social need. This study was approved by the Northwell Health Institutional Review Board. The institutional review board waived the need for informed consent since the screening and referral process was considered a standard of care at the clinic. We followed the Strengthening the Reporting of Observational Studies in Epidemiology (STROBE) reporting guideline.

### SDOH Screening and Referral Program

All caregivers of patients younger than 17 years arriving for a well-child visit are given our Family Wellness Screen (SDOH screen), a standardized paper form of validated and nonvalidated questions to screen for a comprehensive array of unmet social needs by a patient navigator.^16^ Every patient is given the SDOH screen to ensure that the caregiver does not feel singled out, and it is voluntary to complete. After triage, the patient navigator assists with completion as needed and instructs the caregiver to review the results with their physician. The physician then reviews the caregiver’s responses during the clinical encounter and assesses whether the family discloses social needs and desires assistance. The caregiver can accept or decline help by answering the survey question, “Would you be interested in getting help from our practice for anything above?” If a caregiver selects yes to the question, the patient navigator follows up with an intake to gather further information about the patient’s needs. This follow-up is essential to ensure that the screen is patient- and family-centered and allows for shared decision-making.^17^ If the caregiver desires assistance with emergent needs (eg, domestic violence), the physician elicits in-person, real-time patient navigation. For nonemergent social needs, the patient navigator reaches out within 1 to 2 days to conduct an intake, creates a plan, researches resources, and makes referrals to our partner community-based organizations on behalf of the patient. The patient is provided with the resource via phone call and/or email by a patient navigator and notified to expect contact from the referral source.

### Measures and Covariates

The primary outcome was acceptance of community resource assistance among patients with at least 1 unmet social need. We obtained self-reported demographic and social characteristics of the child and caregiver through the SDOH screen.^16^ Demographics included child age, caregiver age and relationship to the child, language spoken at home, child’s race and ethnicity, child nativity, caregiver nativity, and caregiver highest education level. We categorized race and ethnicity as Asian, Pacific Islander, or Native Hawaiian; Black; Latino or Hispanic; White; other (American Indian or Alaska Native, multiracial, multiethnic, and other group), and missing. Race and ethnicity were reported as a variable because of racial and ethnic differences in health care use and the prevalence of social needs. The SDOH screen identified unmet needs, including public benefits, finances, food insecurity, material needs, transportation, childcare, depression, experiences of abuse, medicolegal assistance, unfair job termination, employment, education, health literacy, health insurance, and discrimination. The questions and variables were selected after a thorough review of several previously published tools used for universal SDOH screening of families within health care practices.^16^ We excluded screens in which social support was the only positive response.

### Statistical Analysis

The data analysis was performed from November 18, 2019, through December 17, 2019. The data review was finalized December 20, 2023. We used frequencies and percentages to describe categorical variables by the primary outcome: acceptance of referral services (yes/no). For nonnormally distributed continuous variables, we present the median and interquartile range. Differences between study variables and acceptance of referral services were assessed using the χ^2^ test for categorical variables and the Wilcoxon rank sum test for continuous variables. A 2-sided *P* < .05 was considered to be statistically significant.

We ran multivariable Poisson regression models to evaluate the associations between unmet needs and acceptance of referral services. Because of the large number of items of unmet needs and the high correlations among them, we first performed a categorical principal component analysis,^22^ which reduced the number of items to a few principal components that accounted for the majority of the variance due to those items. Since each item was binary, a tetrachoric correlation matrix was constructed to determine the pairwise associations among need items, excluding pairwise missing observations. This method assumes that each need is manifested from an underlying continuous latent trait. Based on this correlation matrix, the principal component analysis used an oblique rotation because factors were expected to be correlated. The number of components kept was determined by evaluating a scree plot, the proportion of variance retained (73.5%), and eigenvalues of 1 or less. After classifying items on the basis of factor loadings greater than 0.5, 4 principal components were retained and corresponded to items representing basic needs, stress, challenges to economic mobility, and marginalization. The labels of each of the components were selected by the study team based on the needs questions within each component. Principal component scores were then generated by summing the observed items weighted by their factor loading for each component. For each principal component score, an increase in score represented an increase in unmet needs.

We used Poisson regression to model the ratio of probabilities directly instead of using logistic regression because when the outcome is not rare, odds ratios overestimate prevalence ratios (PRs). If a factor had low frequencies (eg, <10 for child nativity), it was not included. The model was also performed with interactions between social support and each principal component score to test whether social support modified the association between scores and acceptance of referral services. Significant interaction effects (*P* < .05) were identified and kept in the model. We reported adjusted PRs, 95% CIs, and *P* values. All analyses were conducted using SAS, version 9.4 (SAS Institute Inc).

Some data were missing for the outcome, including accepting referral services (n = 95 [12.5%]) and the principal components (n = 85 [11.2%]). Missing data for demographic variables were included by retaining a missing category and used in the analysis. The analysis was then performed on complete cases (n = 592 [78.1%]). Imputation was not used because data were likely not missing at random. To understand the potential biases in results using complete case analysis, distributions of complete and incomplete cases were compared (eTable in Supplement 1).

## Results

Of the 758 caregivers who participated in the study, the majority were mothers (614 [81.0%] vs 102 fathers [13.5%] and 10 other caregivers [1.3%]); median caregiver age was 34 years (IQR, 29-40 years). The median age of children was 23 months (IQR, 4-70 months), and 163 were of Asian, Pacific Islander, or Native Hawaiian (21.5%); 213 of Black (28.1%); 156 of Latino or Hispanic (20.6%); 37 of White (4.9%); and 122 of other (16.1%) race and ethnicity (Table 1). Among families with at least 1 unmet social need, 348 (45.9%) accepted referral to services. A higher proportion of caregivers whose children were White (24 [64.9%]) was likely to accept help compared with caregivers whose children were of Asian, Pacific Islander, or Native Hawaiian (88 [54.0%]); Black (70 [32.9%]); Latino or Hispanic (50 [32.1%]); or other race and ethnicity (55 [45.1%]). Additionally, a higher proportion of caregivers with at least a college education (204 of 423 [48.2%] vs 64 of 218 [29.4%] with a high school diploma or less or General Educational Development) was less likely to accept help.

**Table 1.** Sociodemographic Characteristics of Families Who Did and Did Not Accept Assistance

Characteristic	Families, No. (%)				*P* value
	Total	Accepted assistance (n = 348)	Did not accept assistance (n = 315)	Missing (n = 95)	
Child age, median (IQR), mo	23 (4-70)	22 (4-70)	23 (6-74)	24 (2-62)	.45^a^
Child race and ethnicity					
Asian, Pacific Islander, or Native Hawaiian	163 (21.5)	57 (16.4)	88 (27.9)	18 (19.0)	<.001
Black	213 (28.1)	114 (32.8)	70 (22.2)	29 (30.5)	
Latino or Hispanic	156 (20.6)	89 (25.6)	50 (15.9)	17 (17.9)	
White	37 (4.9)	8 (2.3)	24 (7.6)	5 (5.3)	
Other^b^	122 (16.1)	55 (15.8)	55 (17.5)	12 (12.6)	
Missing^c^	67 (8.8)	25 (7.2)	28 (8.9)	14 (14.7)	
Caregiver age, median (IQR), y	34 (29-40)	34 (29-39)	34 (29-40)	36 (30-41)	.72
Caregiver education					
High school diploma or less or GED	218 (28.8)	131 (37.6)	64 (20.3)	23 (24.2)	<.001
College, graduate school, professional school	423 (55.8)	164 (47.1)	204 (64.8)	55 (57.9)	
Missing^d^	117 (15.4)	53 (15.2)	47 (14.9)	17 (17.9)	
Caregiver nativity					
Foreign born	414 (54.6)	196 (56.3)	171 (54.3)	47 (49.5)	.79
US born	272 (35.9)	124 (35.6)	113 (35.9)	35 (36.8)	
Missing^c^	72 (9.5)	28 (8.1)	31 (9.8)	13 (13.7)	
Child nativity					
Foreign born	19 (2.5)	17 (4.9)	1 (0.3)	1 (1.1)	<.001
US born	669 (88.3)	302 (86.8)	286 (90.8)	81 (85.3)	
Missing^c^	70 (9.2)	29 (8.3)	28 (8.9)	13 (13.7)	
Caregiver relationship to child					
Mother	614 (81.0)	291 (83.6)	245 (77.8)	78 (82.1)	.07
Father	102 (13.5)	40 (11.5)	51 (16.19)	11 (11.6)	
Other^e^	10 (1.3)	8 (2.3)	2 (0.6)	0	
Missing^c^	32 (4.2)	9 (2.6)	17 (5.4)	6 (6.3)	
Language					
Speaks English very well	578 (76.3)	254 (73.0)	250 (79.4)	74 (77.9)	.001
Other	94 (12.4)	62 (17.8)	26 (8.3)	6 (6.3)	
Missing	86 (11.4)	32 (9.2)	39 (12.4)	15 (15.8)	

Abbreviation: GED, General Educational Development.

^a^ *P* value from Wilcoxon rank sum test.

^b^ Other race and ethnicity included Native American, multiracial, multiethnic, and other group.

^c^ *P* value from χ^2^ test, excluding missing sociodemographic values.

^d^ *P* value from χ^2^ test, including missing sociodemographic values as a separate category.

The principal component analysis yielded 4 principal components corresponding to items representing basic needs, stress, challenges to economic mobility, and marginalization. An increase in a component score represented an increase in unmet needs for that component. The factor loadings after rotation, which represent the correlation between each item and each component, are presented in Table 2. The distribution of the component scores was different for caregivers who accepted and did not accept assistance (Table 3; eFigure in Supplement 1). There was very low variability in scores among caregivers who did not accept assistance. Among the 315 caregivers who did not accept help, 219 (69.5%) did not endorse any of the need items that were considered for the principal component analysis, which would cause their component scores to be the same. Caregivers who accepted referral services had a higher median basic needs score (0.08 [IQR, −0.39 to 1.17] vs −0.57 [IQR, −0.57 to −0.57]), challenges to economic mobility score (0.05 [IQR, −0.37 to 0.69] vs −0.33 [IQR, −0.33 to −0.33]), and marginalization score (−0.23 [IQR, −0.58 to 0.85] vs −0.37 [IQR, −0.37 to −0.37]), indicating higher needs than those who did not accept referral services.

**Table 2.** Rotated Factor Pattern Identified by Categorical Principal Component Analysis of 15 Unmet Needs

Component and item	Basic needs	Stress	Challenges to economic mobility	Marginalization
**Basic needs**				
Do you want help getting public benefits: food stamps (SNAP), WIC, welfare, or disability (SSI)?	0.875	−0.045	−0.184	0.126
Do you have trouble paying your bills (rent, gas, medical, electricity, water, telephone)?	0.869	0.223	−0.051	−0.099
Do you worry that you would run out of food before you got money to buy more?	0.826	0.12	0.055	0.039
Do you or your child need clothing, diapers, car seats, school supplies, or other items	0.824	0.073	0.140	−0.119
Do problems with transportation stop you from going to medical visits or getting medications?	0.626	−0.150	0.153	0.266
Do you need help getting child care, care for an elderly or sick adult, or more school services for your child?	0.503	−0.066	0.344	0.090
**Stress**				
Have you often felt down, depressed, or hopeless or had little interest or pleasure in doing things?	0.227	0.787	0.029	−0.029
Do you feel unsafe, or has anyone ever hurt/humiliated you or tried to control what you do?	−0.039	0.727	0.243	0.231
Do you need help from a lawyer with housing, immigration, public benefits, or other problems?	0.233	0.601	0.365	−0.012
**Challenges to economic mobility**				
Do you feel as though you’ve been unfairly fired from your job?	−0.024	0.060	0.845	−0.013
Do you need help finding a job?	0.252	0.132	0.612	0.020
As an adult, have you wanted more education but were unable to get it?	−0.040	0.181	0.510	0.456
**Marginalization**				
Do you ever need help understanding what your doctor tells you or reading health information?	0.060	0.174	−0.029	0.770
Do you need help getting health or dental insurance?	0.236	−0.215	0.237	0.717
Have you often felt treated or judged unfairly because of your race, ethnicity, religion, or sexual orientation?	0.011	0.495	−0.240	0.567

Abbreviations: SNAP, Supplemental Nutrition Assistance Program; SSI, Supplemental Security Income; WIC, Women, Infants, and Children.

**Table 3.** Prevalence of Needs of Families Who Did and Did Not Accept Assistance

Needs category and items	Total families, No. (%)	Families accepting help, No. (%)			*P* value^a^
		Yes (n = 348)	No (n = 315)	Missing (n = 95)	
**Basic needs**					
Paying bills					
No	590 (77.8)	211 (60.6)	301 (95.6)	78 (82.1)	<.001
Yes	150 (19.8)	126 (36.2)	10 (3.2)	14 (14.7)	
Missing	18 (2.4)	11 (3.2)	4 (1.3)	3 (3.2)	
Food insecurity					
No	620 (81.8)	228 (65.5)	311 (98.7)	81 (85.3)	<.001
Yes	126 (16.6)	111 (31.9)	3 (1.0)	12 (12.6)	
Missing	12 (1.6)	9 (2.6)	1 (0.3)	2 (2.1)	
Clothing					
No	597 (78.8)	215 (61.8)	303 (96.2)	79 (83.2)	<.001
Yes	150 (19.8)	129 (37.1)	10 (3.2)	11 (11.6)	
Missing	11 (1.5)	4 (1.2)	2 (0.6)	5 (5.3)	
Public benefits					
No	551 (72.7)	185 (53.2)	296 (94.0)	70 (73.7)	<.001
Yes	194 (25.6)	156 (44.8)	18 (5.7)	20 (21.1)	
Missing	13 (1.7)	7 (2.0)	1 (0.3)	5 (5.3)	
Transportation					
No	668 (88.1)	274 (78.7)	309 (98.1)	85 (89.5)	<.001
Yes	79 (10.4)	69 (19.8)	4 (1.3)	6 (6.3)	
Missing	11 (1.5)	5 (1.4)	2 (0.6)	4 (4.2)	
Child care					
No	611 (80.6)	227 (65.2)	309 (98.1)	75 (79.0)	<.001
Yes	131 (17.3)	112 (32.2)	6 (1.9)	13 (13.7)	
Missing	16 (2.1)	9 (2.6)	0	7 (7.4)	
**Stress**					
Hopeless					
No	663 (87.5)	269 (77.3)	307 (97.5)	87 (91.6)	<.001
Yes	83 (11.0)	71 (20.4)	7 (2.2)	5 (5.3)	
Missing	12 (1.6)	8 (2.3)	1 (0.3)	3 (3.2)	
Safety					
No	721 (95.1)	316 (90.8)	314 (99.7)	91 (95.8)	<.001
Yes	25 (3.3)	23 (6.6)	0	2 (2.1)	
Missing	12 (1.6)	9 (2.6)	1 (0.3)	2 (2.1)	
Medical legal need					
No	639 (84.3)	238 (68.4)	313 (99.4)	88 (92.6)	<.001
Yes	109 (14.4)	104 (29.9)	1 (0.3)	4 (4.2)	
Missing	10 (1.3)	6 (1.7)	1 (0.3)	3 (3.2)	
**Challenges to economic mobility**					
Job discrimination					
No	714 (94.2)	315 (90.5)	310 (98.4)	89 (93.7)	<.001
Yes	23 (3.0)	19 (5.5)	3 (1.0)	1 (1.1)	
Missing	21 (2.8)	14 (4.0)	2 (0.6)	5 (5.3)	
Employment					
No	620 (81.8)	229 (65.8)	309 (98.1)	82 (86.3)	<.001
Yes	129 (17.0)	115 (33.1)	6 (1.9)	8 (8.4)	
Missing	9 (1.2)	4 (1.2)	0	5 (5.3)	
More education					
No	540 (71.2)	189 (54.3)	275 (87.3)	76 (80.0)	<.001
Yes	202 (26.7)	150 (43.1)	38 (12.1)	14 (14.7)	
Missing	16 (2.1)	9 (2.6)	2 (0.6)	5 (5.3)	
**Marginalization**					
Health literacy					
No	682 (90.0)	288 (82.8)	304 (96.5)	90 (94.7)	<.001
Yes	69 (9.1)	54 (15.5)	11 (3.5)	4 (4.2)	
Missing	7 (0.9)	6 (1.7)	0	1 (1.1)	
Health insurance					
No	673 (88.8)	277 (79.6)	309 (98.1)	87 (91.6)	<.001
Yes	75 (9.9)	66 (19.0)	6 (1.9)	3 (3.2)	
Missing	10 (1.3)	5 (1.4)	0	5 (5.3)	
Discrimination					
No	680 (89.7)	296 (85.1)	296 (94.0)	88 (92.6)	.001
Yes	64 (8.4)	44 (12.6)	17 (5.4)	3 (3.2)	
Missing	14 (1.9)	8 (2.3)	2 (0.6)	4 (4.2)	
**Other items not grouped**					
Social support					
No	436 (57.5)	128 (36.8)	244 (77.5)	64 (67.4)	<.001
Yes	312 (41.2)	213 (61.2)	70 (22.2)	29 (30.5)	
Missing	10 (1.3)	7 (2.0)	1 (0.3)	2 (2.1)	
Housing					
Other	62 (8.2)	47 (13.5)	9 (2.9)	6 (6.3)	<.001
Private house or apartment	652 (86.0)	282 (81.0)	287 (91.1)	83 (87.4)	
Missing	44 (5.8)	19 (5.5)	19 (6.0)	6 (6.3)	
Housing safety					
No	558 (73.6)	277 (79.6)	281 (89.2)	0	<.001
Yes	43 (5.7)	40 (11.5)	3 (1.0)	0	
Missing	157 (20.7)	31 (8.9)	31 (9.8)	95 (100)	
Housing security					
No	560 (73.9)	236 (67.8)	258 (81.9)	66 (69.5)	<.001
Yes	85 (11.2)	60 (17.2)	20 (6.4)	5 (5.3)	
Missing	113 (14.9)	52 (14.9)	37 (11.8)	24 (25.3)	
Multiple needs					
No	426 (56.2)	78 (22.4)	286 (90.8)	62 (65.3)	<.001
Yes	327 (43.1)	270 (77.6)	27 (8.6)	30 (31.6)	
Missing	5 (0.7)	0	2 (0.6)	3 (3.2)	
**Component scores**					
Basic needs score, median (IQR)	NA	0.08 (−0.39 to 1.17)	−0.57 (−0.57 to −0.57)	−0.57 (−0.57 to 0.11)	<.001
Stress score, median (IQR)	NA	−0.24 (−0.48 to 0.63)	−0.26 (−0.26 to −0.26)	−0.26 (−0.26 to −0.26)	.11
Challenges to economic mobility score, median (IQR)	NA	0.05 (−0.37 to 0.69)	−0.33 (−0.33 to −0.33)	−0.33 (−0.33 to −0.33)	<.001
Marginalized score, median (IQR)	NA	−0.23 (−0.58 to 0.85)	−0.37 (−0.37 to −0.37)	−0.37 (−0.37 to −0.33)	<.001
**No. of needs items (n = 15)**					
Median (IQR)	1 (0 to 3)	3 (2 to 6)	0 (0 to 1)	1 (0 to 2)	<.0001^b^
0	277 (36.5)	12 (3.5)	219 (69.5)	46 (48.4)	<.0001
1	165 (21.8)	73 (21.0)	69 (21.9)	23 (24.2)	
2	82 (10.8)	59 (17.0)	15 (4.8)	8 (8.4)	
3	66 (8.7)	49 (14.1)	9 (2.9)	8 (8.4)	
≥4	168 (22.2)	155 (44.5)	3 (1.0)	10 (10.5)	
**No. of needs items (n = 19)**					
Median (IQR)	4 (4 to 6)	6 (4 to 8)	4 (4 to 4)	4 (3 to 5)	<.0001^b^
1	18 (2.4)	5 (1.4)	10 (3.2)	3 (3.2)	<.0001
2	34 (4.5)	9 (2.6)	16 (5.1)	9 (9.5)	
3	78 (10.3)	14 (4.0)	48 (15.2)	16 (16.8)	
≥4	628 (82.9)	320 (92.0)	241 (76.5)	67 (70.5)	

^a^ *P* value from χ^2^ test unless otherwise noted.

^b^ *P* value from Wilcoxon rank sum test.

Next, we ran multivariable Poisson regression models, which showed each principal component score and interest in referral services (Table 4). Caregivers with basic needs (adjusted PR, 5.56; 95% CI, 3.33-10.00), stress (adjusted PR, 1.75; 95% CI, 1.43-2.17), challenges to economic mobility (adjusted PR, 2.17; 95% CI, 1.67-2.86), or marginalization (adjusted PR, 1.41; 95% CI, 1.15-1.72) were more likely to accept referrals to social services. In addition, Black (adjusted PR, 1.23; 95% CI, 1.01-1.49) and other (adjusted PR, 1.22; 95% CI, 1.01-1.47) race and ethnicity were associated with acceptance of referral services. Housing-related items (living situation, housing stability, and safety) were not associated with acceptance. When including the interaction effect of social support and each principal component score, there was a differential effect of the marginalization component and interest in referral services depending on the presence or lack of social support (*P* for interaction = .04). Among caregivers without social support, a 1-unit increase in marginalization score was associated with an increased prevalence of accepting referral services (adjusted PR, 1.85; 95% CI, 1.32-2.63), whereas for those with social support, marginalization was not associated with accepting services (adjusted PR, 1.18; 95% CI, 0.91-1.54). Interestingly, social support (adjusted PR, 0.91; 95% CI, 0.72-1.14) was not associated with accepting services in the model with main effects only.

**Table 4.** Multivariable Poisson Regression Models to Examine the Association Between Each Needs Component Score and Accepting Referral

Effect	Main effects model		Main effects plus interaction effects model	
	Adjusted PR (95% CI)	*P* value	Adjusted PR (95% CI)	*P* value
Component 1: basic needs	5.56 (3.33-10.00)	<.001	5.56 (3.33-9.09)	<.001
Component 2: stress	1.75 (1.43-2.17)	<.001	1.69 (1.39-2.08)	<.001
Component 3: challenges to economic mobility	2.17 (1.67-2.86)	<.001	2.08 (1.64-2.70)	<.001
Component 4: marginalization	1.41 (1.15-1.72)	.001	NA	NA
Among social support, no	NA	NA	1.85 (1.32-2.63)	<.001
Among social support, yes	NA	NA	1.18 (0.91-1.54)	.21
Social support, yes vs no	0.91 (0.72-1.14)	.42	NA	NA
Child age, mo	1.00 (1.00-1.00)	.89	1.00 (1.00-1.00)	.70
Child race and ethnicity				
Asian, Pacific Islander, or Native Hawaiian	1.15 (0.95-1.37)	.14	1.12 (0.95-1.33)	.18
Black	1.23 (1.01-1.49)	.04	1.20 (1.00-1.47)	.050
Latino or Hispanic	1.11 (0.88-1.41)	.38	1.09 (0.85-1.37)	.50
White	1 [Reference]	NA	1 [Reference]	NA
Other^a^	1.22 (1.01-1.47)	.04	1.20 (1.00-1.45)	.06
Missing	1.04 (0.81-1.35)	.73	1.03 (0.8-1.32)	.83
Education level				
College, graduate school, professional school	1 [Reference]	NA	1 [Reference]	NA
Missing	1.09 (0.93-1.27)	.27	1.10 (0.93-1.28)	.26
High school diploma or less or GED	1.11 (0.93-1.32)	.25	1.10 (0.93-1.30)	.30
Language				
Speaks English very well	1 [Reference]	NA	1 [Reference]	NA
Missing	0.89 (0.76-1.05)	.19	0.88 (0.75-1.04)	.15
Other	0.85 (0.65-1.10)	.22	0.85 (0.66-1.11)	.25
Relationship to child				
Mother	1 [Reference]	NA	1 [Reference]	NA
Missing	0.95 (0.74-1.22)	.68	0.97 (0.76-1.25)	.82
Father/other	1.01 (0.89-1.15)	.83	1.03 (0.90-1.16)	.68
Living situation				
Private house or apartment	1 [Reference]	NA	1 [Reference]	NA
Missing	0.95 (0.69-1.32)	.76	0.93 (0.68-1.30)	.70
Other	1.11 (0.76-1.61)	.58	1.14 (0.79-1.64)	.49
Safe and stable				
No	1 [Reference]	NA	1 [Reference]	NA
Missing	1.00 (0.83-1.2)	.98	1.01 (0.84-1.22)	.92
Yes	1.09 (0.85-1.39)	.51	1.09 (0.85-1.39)	.51
Housing safety				
No	1 [Reference]	NA	1 [Reference]	NA
Yes	2.63 (0.93-7.14)	.07	2.44 (0.85-7.14)	.10
Missing	1.08 (0.87-1.32)	.52	1.06 (0.87-1.32)	.55

Abbreviations: NA, not applicable; PR, prevalence ratio.

^a^ Other race and ethnicity included Native American, multiracial, multiethnic, and other group.

A comparison of missing and nonmissing cases (eTable in Supplement 1) showed that missing cases were not random. For example, racial and ethnic minority children had higher rates of incomplete cases compared with White children.

## Discussion

This cross-sectional study showed that patient characteristics, specifically child race and ethnicity and the caregiver’s education, were significantly associated with patient families’ interest in assistance. In addition, we observed social support to have interaction effects only if a patient screened for a need that fell in the marginalization score and did not have social support; their family was more likely to accept help.

This study also showed that caregiver declaration of a particular need, whether a basic need, a stress need, a challenge to economic mobility need, or a marginalization need, was associated with a higher prevalence of being interested in assistance with referral services. More specifically, we found that a 1-unit increase in the basic needs principal component score increased the prevalence of accepting help more than 1 unit in the stress or challenges to economic mobility score. Basic needs included what we described as more tangible need items, such as food or clothing, which may have more urgency. While families with intangible needs were interested in seeking help for those items or for services to address such needs, identifying or addressing such needs might be challenging. Help for intangible items may have appeared out of scope to patients in a clinical setting,^11^ so a review of the language used may be needed to discover a clearer way of communicating that the child’s health facility may be able to help with seemingly non–health-related concerns because they affect the overall health of the child.

This study also found that families with a need in the marginalization score who did not have social support were more likely to be interested in assistance. Acceptance of services to address social needs may depend on social support or social capital. Social capital has been defined as features of social organization, such as networks, norms, and social trust, that facilitate coordination and cooperation for mutual benefit.^23^ Social capital may be gained through personal relationships with experts or on a contractual basis, such as paid consultants and services.^24^ We found that nonmaterial needs, which fell into the stress, challenges to economic mobility, and marginalization scores, included needs associated with lack of social capital and were associated with not being interested in assistance compared with needs that fell into the basic needs score. We believe that these nonmaterial needs represent the concept of social capital. Social capital is unequally distributed in society, and it is influenced by race, ethnicity, and gender.^25^ Access to social resources tends to be limited among racial and ethnic minority individuals and women.^25^ Having adequate social capital within their communities may lead to not being interested in getting help from the provided SDOH referral and navigation program. Individuals who have a higher education level may have people in their networks who could provide free or low-cost aid. Moreover, another possibility may be that the degree to which patient families needed help with their social needs was less acute and smaller in magnitude. The screening questionnaires did not quantify the urgency of their needs. In another study,^26^ increased marginalization (ie, more social needs) without social support was associated with no-shows to well-child visits, but the presence of adequate social support negated the association.

Previous studies have shown long-term consequences of stress, challenges to economic mobility,^27^ and marginalization.^28,29^ Kawachi et al^21^ found that the association of income inequality with mortality was mediated by social capital. There is a positive association of social capital with SDOH needs.^20,30^ Investment in programs to increase social capital, such as addressing unmet social needs, has been successful. For example, addressing food insecurity has been associated with improved social and clinical measures. Nutritional support through Women, Infants, and Children and the Canada Prenatal Nutrition Program has been associated with a decreased likelihood of preterm delivery, low birth weight, and fetal death. Providing education about nutritional support programs may improve food security and efficiency.^31,32^ Food prescription programs that provide fresh fruits and vegetables have led to a substantial drop in food insecurity.^33^ Another area of social capital that may garner stronger connections and resources is education. Educational attainment is a powerful determinant of health outcomes as it improves health knowledge, healthy behaviors, and employment opportunities.^11,34^ As educational levels increase, so might social capital. Social capital resources may lead to some educational achievements, yet education may also be a tool for increasing the level of civic participation.^35^

### Limitations

Our study has several limitations. We only included caregivers who completed the SDOH screening questionnaires. Examination of our data showed that a higher percentage of Black families did not complete the SDOH screening. Incompletion of the screen may have arisen from the caregiver’s belief that participating in the referral program may not address their social needs, which could lead to selection bias in which more Black families who were interested in SDOH referrals were to be included in the study. Another possibility is the time frame of the screening, that caregivers who were afraid of public charge may have declined the services. Public charges may penalize individuals seeking legal residence or US citizenship, which may lead to a higher decline in participation in the referral process among immigrants. Moreover, missing data were not part of the exclusion criteria but did get dropped in the analyses. The comparison between missing and nonmissing cases showed that missing cases were not random. Caregivers may have left the option blank because their real response to the question was likely to decline. Finally, the data were collected prior to the COVID-19 pandemic because the SDOH screening was paused due to health safety concerns.

## Conclusions

This cross-sectional study of patient families with at least 1 unmet social need found that child race and ethnicity, caregiver education, and having tangible social needs were associated with interest in receiving referral assistance. Our findings suggest that a culturally sensitive SDOH screening referral program may provide equitable care, especially to racial and ethnic minority families. Even after adjusting for covariates, caregivers who had more social needs, including those of racial and ethnic minority groups, were interested in using the social referral system. Targeting underrepresented populations that have historically been marginalized is necessary to reach a state of equitable health. This study supports the growing body of research intended to address patients’ unmet social needs in clinical settings to achieve health equity. However, more work is needed to convey the available support for unmet social needs and identify interventions to increase use as there are patients with social needs who do not accept referrals to social services.

## Notes

Supplement 1. **eTable.** Comparison of Complete Cases and Incomplete Cases **eFigure.** Distributions of Principal Component Scores by Interest in Accepting Assistance

Supplement 2.
Data Sharing Statement
