# Supplementary material for: Participation in Clinic-Based Referral and Navigation Services Among Families With Social Needs
Source: JAMA Netw Open. 2025 Feb 28;8(2):e250056. doi: 10.1001/jamanetworkopen.2025.0056 (PMC11871544; doi:10.1001/jamanetworkopen.2025.0056)
Supplement: Supplement 1. — eTable. Comparison of Complete Cases and Incomplete Cases eFigure. Distributions of Principal Component Scores by Interest in Accepting Assistance [file jamanetwopen-e250056-s001.pdf]

Supplemental Online Content

Seide A, Uwemedimo OT, Rasul R, et al. Participation in clinic-based referral and navigation services among families with social needs. *JAMA Netw Open*. 2025;8(2):e250056.
doi:10.1001/jamanetworkopen.2025.0056

eTable. Comparison of Complete Cases and Incomplete Cases

eFigure. Distributions of Principal Component Scores by Interest in Accepting Assistance

This supplemental material has been provided by the authors to give readers additional information about their work.

© 2025 Seide A et al. *JAMA Network Open.*

eTable. Comparison of Complete Cases and Incomplete Cases		**Incomplete Cases**		**Complete Cases**		
**VARIABLE**	**CATEGORY**	**n**	**%**	**n**	**%**	**N**
Child Ethnicity	Asian/Pacific Islander/Native Hawaiian	28	16.9	135	22.8	758
	Black	55	33.1	158	26.7	
	Latino/Hispanic	37	22.3	119	20.1	
	Native American/Multiple Race/Ethnicity/Other	21	12.7	101	17.1	
	White	6	3.6	31	5.2	
	Missing	19	11.5	48	8.1	
Language	English/Very well	125	75.3	453	76.5	758
	Other	14	8.4	80	13.5	
	Missing	27	16.3	59	10.0	
Education Level	Equal or less than high school diploma or GED	47	28.3	171	28.9	758
	College/Graduate/Professional School	86	51.8	337	56.9	
	Missing	33	19.9	84	14.2	
Social Support	No	94	60.3	342	57.8	748
	Yes	62	39.7	250	42.2	
Housing Safety	No	20	12.1	28	4.7	758
	Yes	124	74.7	510	86.2	
	Missing	22	13.3	54	9.1	
Relationship to Child	Father/Other	22	13.3	90	15.2	758
	Mother	138	83.1	476	80.4	
	Missing	6	3.6	26	4.4	
Housing	Private House/Apartment	138	83.1	514	86.8	758
	Other	16	9.6	46	7.8	
	Missing	12	7.2	32	5.4	
Housing Stability	No	107	64.5	453	76.5	758
	Yes	22	13.3	63	10.6	
	Missing	37	22.3	76	12.8	
Help From Practice	No	17	23.9	298	50.3	663
	Yes	54	76.1	294	49.7	

© 2025 Seide A et al. *JAMA Network Open.*

eFigure. Distributions of Principal Component Scores by Interest in Accepting Assistance

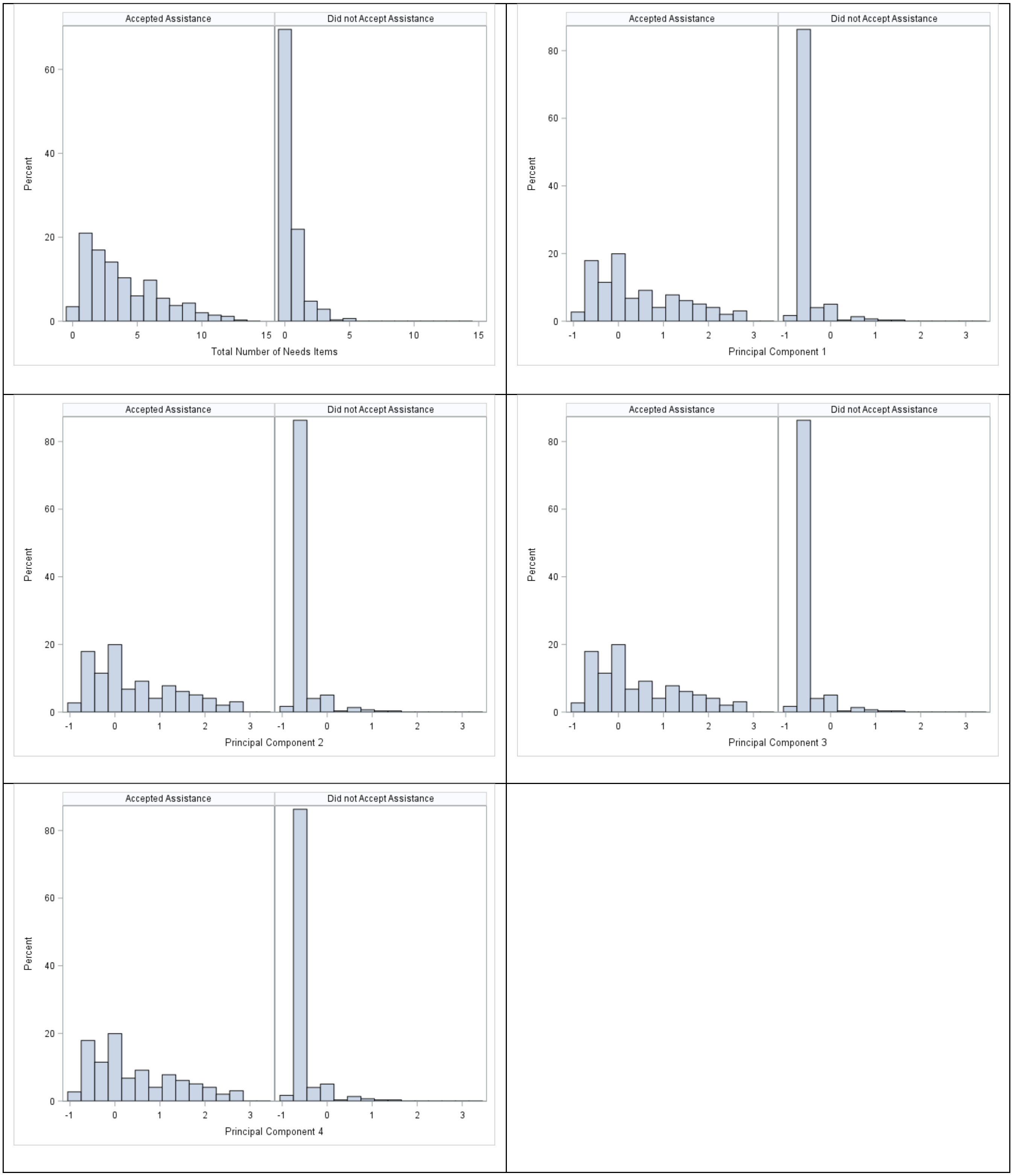

© 2025 Seide A et al. *JAMA Network Open.*
