# Supplementary material for: Participation in Clinic-Based Referral and Navigation Services Among Families With Social Needs
Source: JAMA Netw Open. 2025 Feb 28;8(2):e250056. doi: 10.1001/jamanetworkopen.2025.0056 (PMC11871544; doi:10.1001/jamanetworkopen.2025.0056)
Supplement: Supplement 2. — Data Sharing Statement [file jamanetwopen-e250056-s002.pdf]

Data Sharing Statement

Seide. Participation in Clinic-Based Referral and Navigation Services Among Families With Social Needs. *JAMA Netw Open*. Published February 28, 2025.
doi:10.1001/jamanetworkopen.2025.0056

Data
Data available: No

Additional Information
Explanation for why data not available: Upon request, a limited dataset will be made available for other researchers for the purposes of validation of findings. The limited dataset will include de-identified data relevant to the specific request. Independent research groups can view relevant data to evaluate the extent that data sources support conclusions made by authors in published studies as well as observe additional emergent findings and view supplemental details that might not be included in publications.
